# Causal Association Between Inflammatory Factors and Hypertrophic Scar: A Two‐Sample Mendelian Randomization Study

**DOI:** 10.1111/jocd.70073

**Published:** 2025-02-27

**Authors:** Yanqi Li, Yankun Zhang, Wanchao Wang, Yuge Wang, Hongmei Ai

**Affiliations:** ^1^ Department of Plastic Surgery Emergency General Hospital/National Research Center for Emergency Medicine Beijing China; ^2^ Department of Dermatology and Medical Cosmetology Beijing Chao‐Yang Hospital, Capital Medical University Beijing China

**Keywords:** causal association, CTACK, ETHE1, hypertrophic scar, inflammatory factors, Mendelian randomization

## Abstract

**Background:**

Hypertrophic scars result from abnormal healing following skin injuries.

**Aim:**

To delve deeper into the causal association between inflammatory factors and hypertrophic scars.

**Methods:**

This study utilized genetic data from the FINN cohort and pertinent literature to scrutinize the nexus between a spectrum of inflammatory factors—encompassing IL‐1β, interleukin 1 receptor‐like 1, MCP1, RANTES/CCL5, TNFα, IL‐8, IL‐18, and CTACK/CCL27—and the risk of hypertrophic scarring. Our analytical strategy was based on the inverse variance weighted (IVW) approach, further bolstered by MR‐Egger, weighted median, and weighted mode methods to ensure a comprehensive assessment. The reliability of our findings was rigorously appraised through Cochran's Q test, MR‐Egger regression, MR‐PRESSO, and leave‐one‐out analysis.

**Results:**

The genetic prediction results revealed a significant association between CTACK and hypertrophic scars (OR 1.21, 95% CI 1.05–1.4, *p* = 0.01) using the IVW method, although it was not corroborated by other MR analysis methods. The remaining inflammatory factors did not exhibit significant correlations with the risk of hypertrophic scar formation (all *p* > 0.05). The absence of significant heterogeneity among the IVs was indicated by Cochran's Q test. MR‐Egger and MR‐PRESSO analyses collectively suggested no substantial horizontal pleiotropy influencing the results, except for the relationship between RANTES and hypertrophic scars. Upon exclusion of an outlier, the causal relationship between RANTES and hypertrophic scars was found to be non‐significant.

**Conclusion:**

Our MR analysis supports a causal association between CTACK and hypertrophic scars, enhancing our understanding of scar formation and suggesting potential targeted therapeutic strategies for treatment.

## 1 Introduction

Hypertrophic scars, a common sequela in burn patients, affect an estimated 70% of individuals, manifesting as an overgrowth of tissue that is both raised and thick at the site of healed wounds [1]. Surgical incisions are also prone to this condition, with 34%–64% of patients developing hypertrophic scars post‐surgery [2]. The etiology of hypertrophic scars is multifactorial, encompassing local wound stress, systemic conditions such as hypertension, genetic predispositions like single‐nucleotide polymorphisms, and even lifestyle factors. The management of hypertrophic scar volume is essential for improving patient comfort and reducing discomfort and itch. A tailored approach to intervention is paramount, with clinical options ranging from surgical procedures to radiotherapy and conservative treatments including silicone gel sheets, tape, and topical or injectable medications [3]. It is crucial to individualize treatment to prevent adverse outcomes like skin atrophy, telangiectasia, pigmentation changes, ulceration, and other skin imperfections [4]. The presence of hypertrophic scars imposes a significant psychological and economic strain on patients, underscoring the necessity for in‐depth research on potential risk factors that can be controlled and the broader implications of this condition.

Hypertrophic scars typically arise from skin injuries and irritations, such as trauma, burns, surgery, and acne. It is noteworthy that superficial injuries not reaching the reticular dermis do not lead to keloids or hypertrophic scars [5]. This suggests that the formation of these abnormal scars is associated with deeper damage affecting the reticular dermis and an ensuing irregular wound healing response characterized by persistent and localized inflammation. The role of inflammatory factors in the pathogenesis of hypertrophic scars has been suggested by several clinical observations, indicating a possible link between the inflammatory response and scar formation. For example, studies have shown increased protein levels of prostaglandin E2, IL‐6, IL‐8, and monocyte chemotactic protein‐1 (MCP‐1) in hypertrophic scar fibroblasts compared to normal skin, as measured by ELISA assays. When comparing normal and hypertrophic scar fibroblasts, significant upregulation of MyD88, IL‐6, IL‐8, and MCP‐1 was observed upon LPS stimulation [6]. Similarly, increased IL‐6 expression and secretion in fibroblasts derived from hypertrophic burn scars (HBSs) have been reported, suggesting a role for inflammatory cytokines in the pathogenesis of HBSs [7]. These findings point to a substantial correlation between inflammatory mediators and hypertrophic scar development. However, establishing causality is complex due to potential confounders and the risk of reverse causation.

Mendelian randomization, a novel statistical method, assesses causal relationships by using genetic variations as instrumental variables [8]. This approach helps to counteract the effects of confounding factors and reverse causation due to the random distribution of genetic traits during fertilization, thereby overcoming several limitations inherent in observational studies [8, 9]. The current study seeks to elucidate the relationship between inflammatory factors and hypertrophic scars, with the goal of deepening our comprehension of the mechanisms involved and pinpointing new therapeutic avenues for treating this intricate skin condition.

## 2 Methods

### 2.1 Study Design

**FIGURE 1:**
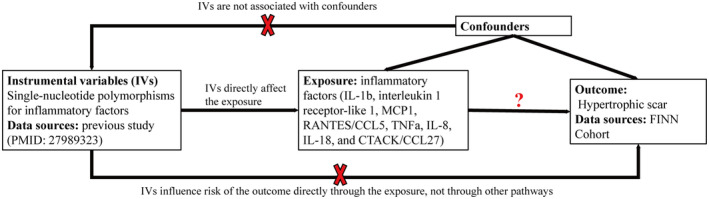
Workflow of MR study revealing causal relationship between inflammatory factors and hypertrophic scar. MR, Mendelian randomization.

Our Mendelian randomization study, as depicted in Figure 1, adheres to the MR‐STROBE (Appendix S1) guidelines [10] and is designed to investigate the potential causal relationships between inflammatory factors and hypertrophic scars. The two‐sample MR approach employed in this study is predicated on three key assumptions: firstly, that the instrumental variables (IVs) are robustly related to the exposure of interest; secondly, that the IVs are not confounded by factors that influence both exposure and outcome; and thirdly, that the IVs influence the outcome solely through the exposure, excluding direct causal pathways [11].

### 2.2 Data Sources

We obtained genetic summary data for hypertrophic scars from the FINN cohort, comprising 1641 cases and 385,509 controls. Data on inflammatory factors were sourced from a previous study [12], encompassing IL‐1β, interleukin 1 receptor‐like 1, MCP1, RANTES/CCL5, tumor necrosis factor alpha (TNFα), IL‐8, IL‐18, and CTACK/CCL27. Comprehensive summaries are provided in Table S1. Given the use of publicly available data, no ethical clearance was sought.

### 2.3 Instrumental Variable Selection

The IVs for this research were chosen based on rigorous standards: initially, a *p*‐value threshold of < 5 × 10^−8^ was applied, later adjusted to < 5 × 10^−6^ to broaden the selection of candidate SNPs related to inflammatory factors. SNPs with a minor allele frequency above 0.01 were included, excluding those in high linkage disequilibrium (*R* ^2^ < 0.001, window size of 10 000 kb). Proxy SNPs with significant linkage disequilibrium (*R* ^2^ > 0.8) were utilized when the IV was not present in the outcome's summary data. The strength of each IV was evaluated using the F‐statistic (*F* = *R* ^2^ × (*N*−2)/(1−*R* ^2^)), with a threshold > 10 to mitigate weak instrument bias [13].

### 2.4 Mendelian Randomization Analysis

We utilized the inverse variance weighted (IVW) method, yielding odds ratios (OR) and 95% confidence intervals (CI), to determine the potential causal links between inflammatory factors and the development of hypertrophic scars [14]. Supplementary MR analyses were conducted using the MR‐Egger, weighted median, and weighted mode methods. All of these analyses were executed using R version 4.3.2, specifically with the “Two‐sample MR” package, and the results were graphically represented through forest plots, scatter plots, and funnel plots to facilitate a clear interpretation of the data.

### 2.5 Sensitivity Analysis

To ensure the reliability of our MR analysis, we conducted a thorough sensitivity analysis. Heterogeneity among IVs was assessed using Cochran's *Q* test, which is integrated within the IVW analysis framework. Furthermore, to address the possibility of genetic pleiotropy, which refers to a single genetic variant affecting multiple traits, we employed MR‐Egger regression. This method is designed to detect and adjust for pleiotropic effects that could skew the estimated causal relationships. Additionally, we utilized the MR pleiotropy residual sum and outlier (MR‐PRESSO) analysis to identify any outliers among the IVs. The MR‐PRESSO technique not only pinpoints these outliers but also allows for their exclusion, thereby refining the causal association estimate. To further substantiate our findings, we performed a leave‐one‐out sensitivity analysis [15]. This involved iteratively removing one IV at a time from the analysis and recalculating the effect estimates to verify that no single IV was disproportionately influencing the results. This comprehensive approach ensures that our conclusions are robust and not susceptible to the influence of outliers or other anomalies in the data.

## 3 Results

### 3.1 Instrumental Variable Selection

After implementing a series of quality control measures, the selection of IVs for the MR analysis was meticulously refined. Our study focused on exposure to various inflammatory factors, including IL‐1β, interleukin 1 receptor‐like 1, MCP1, RANTES/CCL5, TNFα, IL‐8, IL‐18, and CTACK/CCL27. The number of IVs identified for each protein is 5, 25, 15, 11, 5, 4, 19, and 12, respectively, as detailed in Table S2. Notably, for IL‐1 receptor‐like 1, IL‐18, and CTACK/CCL27 as exposures, information for 2 SNPs (rs56144230, rs532436), 1 SNP (rs11700536), and 1 SNP (rs145902143) was not matched in the summary data. For IL‐18, two palindromic SNPs (rs1656939, rs4414903) were excluded. The F‐statistics for all selected IVs significantly surpassed the threshold of 10, highlighting the robustness of the instruments used in this two‐sample MR study.

### 3.2 The Casual Effect of Inflammatory Factors on Hypertrophic Scars

**FIGURE 2:**
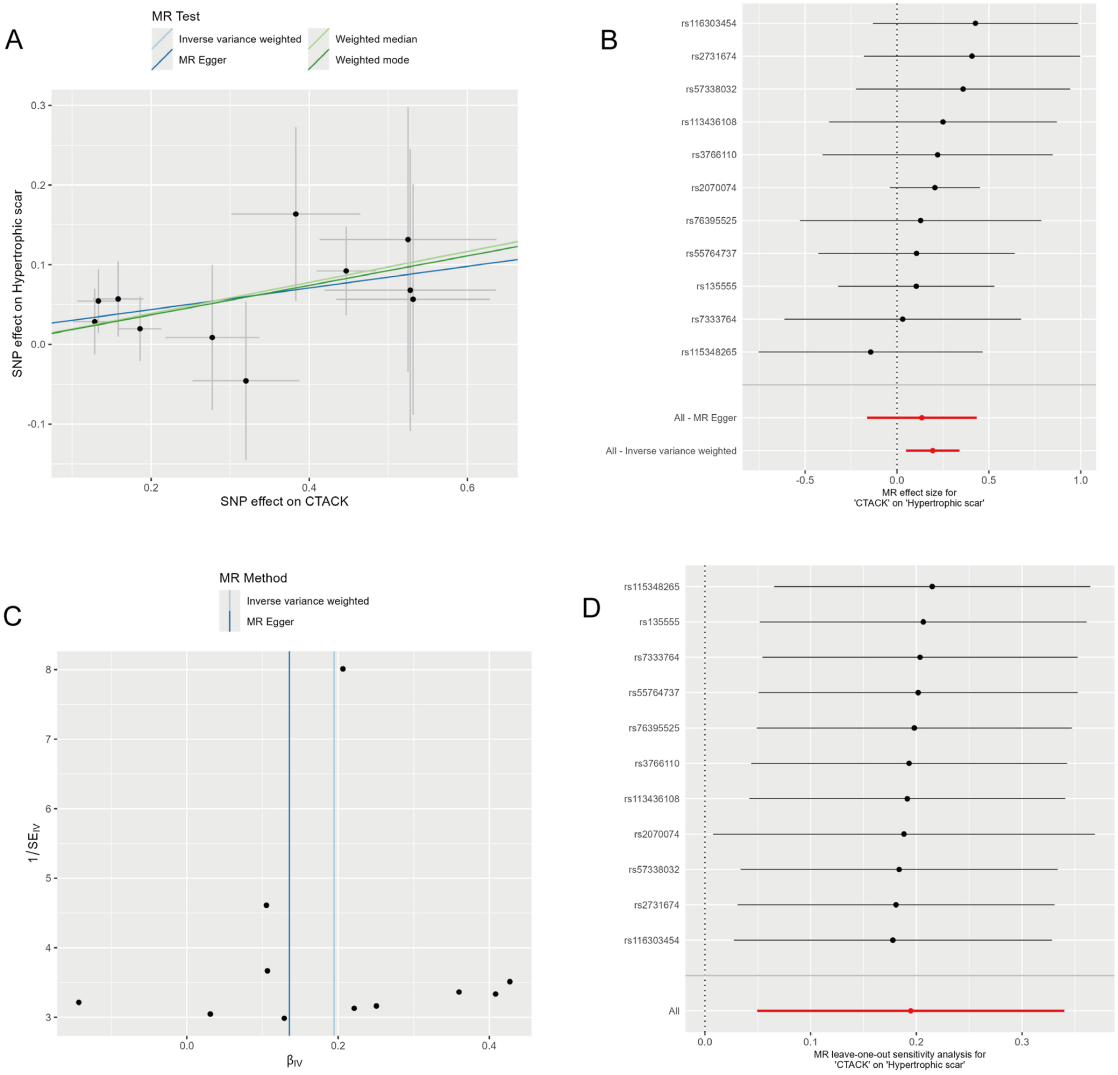
The causal relationship between CTACK and hypertrophic scar by MR analysis. (A) Scatter plot of MR models for potential relationship between CTACK and hypertrophic scar. (B) Forest plot of MR effect size for potential relationship between CTACK and hypertrophic scar. (C) Funnel plot of IVW model and MR‐Egger model for potential relationship between CTACK and hypertrophic scar. (D) MR leave‐one‐out sensitivity analysis for potential relationship between CTACK and hypertrophic scar. MR, Mendelian randomization.

Our systematic investigation examining the potential causal links between eight inflammatory markers and hypertrophic scarring is presented in Table 1. The IVW method revealed a notable association between CTACK and hypertrophic scar (OR 1.21, 95% CI 1.05–1.4, *p* = 0.01), indicating that CTACK is a risk factor for hypertrophic scar. The scatter plots in Figure 2A illustrate the correlation between CTACK and hypertrophic scars, and Figure 2B provide a visual representation of the MR findings, clarifying the relationship. However, this association was not corroborated by other methods, such as the weighted median method (OR 1.21, 95% CI 1.00–1.47, *p* = 0.05), MR Egger method (OR 1.15, 95% CI 0.85–1.54, *p* = 0.4), or weighted mode analysis (OR 1.2, 95% CI 0.96–1.51, *p* = 0.14). No other significant correlations were identified between the remaining inflammatory factors and the risk of hypertrophic scar (all *p* > 0.05).

**TABLE 1:** MR Analysis of inflammatory factors and risk of hypertrophic scar.

Exposure	Outcome	N.SNPs	Methods	OR (95% CI)	*p*
IL‐1β	Hypertrophic scar	5	Inverse variance weighted	1.12 (0.84–1.49)	0.45
IL‐1β		5	MR Egger	0.71 (0.37–1.37)	0.38
IL‐1β		5	Weighted median	1.05 (0.74–1.49)	0.79
IL‐1β		5	Weighted mode	1.05 (0.76–1.45)	0.8
IL1RL1	Hypertrophic scar	23	Inverse variance weighted	0.95 (0.84–1.07)	0.38
IL1RL1		23	MR Egger	0.97 (0.8–1.17)	0.74
IL1RL1		23	Weighted median	0.92 (0.79–1.08)	0.31
IL1RL1		23	Weighted mode	0.93 (0.79–1.08)	0.34
MCP1	Hypertrophic scar	15	Inverse variance weighted	0.89 (0.75–1.05)	0.16
MCP1		15	MR Egger	0.94 (0.66–1.34)	0.72
MCP1		15	Weighted median	0.95 (0.77–1.17)	0.62
MCP1		15	Weighted mode	0.97 (0.75–1.24)	0.79
RANTES	Hypertrophic scar	10	Inverse variance weighted	0.88 (0.71–1.1)	0.26
RANTES		10	MR Egger	0.94 (0.51–1.75)	0.85
RANTES		10	Weighted median	0.96 (0.76–1.23)	0.75
RANTES		10	Weighted mode	1.1 (0.72–1.69)	0.66
TNFα	Hypertrophic scar	5	Inverse variance weighted	1.01 (0.78–1.29)	0.96
TNFα		5	MR Egger	1.24 (0.81–1.88)	0.4
TNFα		5	Weighted median	1.04 (0.76–1.43)	0.79
TNFα		5	Weighted mode	1.1 (0.73–1.65)	0.67
IL‐8	Hypertrophic scar	4	Inverse variance weighted	1.08 (0.77–1.52)	0.66
IL‐8		4	MR Egger	2.18 (0.98–4.85)	0.2
IL‐8		4	Weighted median	1 (0.69–1.45)	0.99
IL‐8		4	Weighted mode	0.96 (0.59–1.56)	0.88
IL‐18	Hypertrophic scar	16	Inverse variance weighted	0.94 (0.84–1.06)	0.33
IL‐18		16	MR Egger	0.94 (0.72–1.21)	0.63
IL‐18		16	Weighted median	0.96 (0.82–1.12)	0.62
IL‐18		16	Weighted mode	0.97 (0.79–1.19)	0.8
CTACK	Hypertrophic scar	11	Inverse variance weighted	1.21 (1.05–1.4)	0.01
CTACK		11	MR Egger	1.15 (0.85–1.54)	0.4
CTACK		11	Weighted median	1.21 (1–1.47)	0.05
CTACK		11	Weighted mode	1.2 (0.96–1.51)	0.14

### 3.3 Sensitivity Analysis

To ensure the robustness of our findings, sensitivity analysis was conducted. The Cochran's Q test, integrated within the IVW method, indicated no significant heterogeneity among the IVs (all *p* > 0.05, Table 2). Furthermore, the MR‐Egger intercept test and the MR‐PRESSO analysis were employed to assess the potential for pleiotropy, which is the phenomenon where genetic variants affect the outcome through multiple biological pathways. The results from both methods, detailed in Tables 2 and 3, indicated that our findings were generally free from pleiotropy, with the exception of the relationship involving RANTES and hypertrophic scarring. Specifically, an outlier SNP (rs112072646) was identified in the MR‐PRESSO analysis when RANTES was the exposure variable (Table 3). After its exclusion, the causal relationship between RANTES and hypertrophic scar remained non‐significant (OR 0.881, 95% CI 0.71–1.1, *p* = 0.29), reinforcing the robustness of our findings. The symmetrical distribution of funnel plots in Figure 2C around the estimated effect line indicates no publication bias. Moreover, the leave‐one‐out analysis, visualized in Figure 2D, demonstrated the stability of the association between CTACK and hypertrophic scarring. This analysis involved the sequential removal of each SNP to assess the influence of individual genetic variants on the overall results. The consistency of the observed associations even after the removal of any single SNP underscores the robustness of our findings.

**TABLE 2:** Results of sensitivity analysis of inflammatory factors and hypertrophic scar.

Exposure	Outcome	Heterogeneity		Pleiotropy	
		Q statistic (IVW)	*p*	MR‐Egger intercept	*p*
IL‐1β	Hypertrophic scar	2.67	0.61	0.077	0.23
IL1RL1		15.23	0.85	−0.005	0.77
MCP1		7.8	0.9	−0.009	0.76
RANTES		12.77	0.17	−0.014	0.83
TNFα		1.53	0.82	−0.052	0.32
IL‐8		3.92	0.27	−0.104	0.21
IL‐18		10.23	0.81	0.002	0.96
CTACK		3.27	0.97	0.017	0.67

**TABLE 3:** Results of MRPRESSO analysis of inflammatory factors and hypertrophic scars.

Exposure	Outcome	Raw		Outlier corrected		Global *p*	Number of outliers	Distortion *p*
		OR (CI%)	*p*	OR (CI%)	*p*			
IL‐1β	Hypertrophic scar	1.12 (0.88–1.41)	0.41	NA	NA	0.708	NA	NA
IL1RL1		0.95 (0.85–1.05)	0.31	NA	NA	0.84	NA	NA
MCP1		0.89 (0.79–1.01)	0.08	NA	NA	0.904	NA	NA
RANTES		0.96 (0.75–1.24)	0.78	0.88 (0.71–1.1)	0.29	0.018	1 (rs112072646)	0.664
TNFα		1.01 (0.86–1.18)	0.94	NA	NA	0.85	NA	NA
IL‐8		1.08 (0.77–1.52)	0.69	NA	NA	0.328	NA	NA
IL‐18		0.94 (0.85–1.04)	0.26	NA	NA	0.835	NA	NA
CTACK		1.21 (1.12–1.32)	0	NA	NA	0.973	NA	NA

## 4 Discussion

This study employs an MR approach to delineate the causal nexus between inflammatory factors and hypertrophic scar formation, a condition that poses a significant challenge to burn victims and post‐surgical patients. Our robust two‐sample MR analysis has unearthed a significant association between the chemokine CTACK and hypertrophic scars, as determined by the IVW method. This revelation casts new light on the intricate dynamics between inflammatory processes and the pathogenesis of scar formation. The rigor of our study is underscored by comprehensive sensitivity analyses, including Cochran's Q test, MR‐Egger regression, MR‐PRESSO analysis, and leave‐one‐out sensitivity analysis, all of which have fortified the credibility of our findings.

CTACK, also known as CCL27 or ESkine, is a chemokine that is primarily expressed in skin keratinocytes and plays a pivotal role in the recruitment of skin‐resident lymphocytes, such as T cells and innate lymphoid cells, which carry CCR10—CTACK's sole known receptor [16]. Historically, the CCL27/CCR10 axis has been implicated in the regulation of inflammatory T cell infiltration into the skin [17]. Recent research has expanded our understanding of its function in the wound healing process. For example, research by IInokuma and colleagues has shown that CTACK is not only present in normal skin but also increases in wounded areas, promoting the migration of bone marrow‐derived keratinocytes and speeding up the wound healing process without influencing angiogenesis or keratinocyte proliferation [18]. Additionally, Broek et al. have found CTACK in burn wound exudates, highlighting its importance in wound healing [19].

Moreover, the impaired migration of adipose‐derived stromal cells in high‐glucose conditions, typical of diabetic wounds, can be partially reversed by the addition of CTACK, indicating its potential role in the healing process [20]. Recent studies have shed light on the association between CTACK and hypertrophic scars. For instance, Limandjaja and colleagues have reported a distinct profile of inflammatory cytokine and growth factor secretion among different scar types. Specifically, they observed a reduction in CTACK secretion in hypertrophic and keloid scars when compared to normal skin and normotrophic scars [21]. Our study builds upon these findings by suggesting a causal relationship between CTACK and hypertrophic scars, identifying CTACK as a potential risk factor for hypertrophic scarring. However, the underlying mechanisms remain to be fully elucidated. Given that CTACK is recognized for its role in chemoattracting T‐cells and other immune cells to inflammatory sites, and considering the close link between inflammation and the formation of hypertrophic scars, an elevated presence of CTACK could potentially amplify the inflammatory response. This heightened inflammation may lead to an excessive aggregation of immune cells, which could precipitate hypertrophic scarring. Furthermore, CTACK may also be implicated in the activation and proliferation of fibroblasts, the primary cells accountable for collagen synthesis. Overstimulation of fibroblasts could result in an overabundance of collagen and extracellular matrix components, which are hallmark characteristics of hypertrophic scars. These hypotheses, while plausible, necessitate further research to confirm their validity.

Recent scholarly work has begun to unravel the intricate connections between other inflammatory mediators and hypertrophic scars. Studies by Kwan and colleagues have pointed out that early serum levels of decorin and IL‐1β, as well as later serum levels of TGF‐β1, are indicative of the likelihood of hypertrophic scar formation [22]. This finding underscores the potential role of these biomarkers in the early and late stages of wound healing, respectively. Additionally, research has demonstrated that naringenin can hinder the activation of fibroblasts and the recruitment of inflammatory cells by reducing the expression of pro‐inflammatory cytokines such as TNF‐α, IL‐1β, IL‐6, and TGF‐β1, thus potentially preventing hypertrophic scarring [23]. These findings imply that targeting the inflammatory response could offer a therapeutic strategy. Consistent with these findings, other studies have reported an upregulation of pro‐inflammatory factors like IL‐1α, IL‐1β, IL‐6, and TNF‐α in keloid tissues, which are characterized by a fibro‐proliferative skin disorder analogous to hypertrophic scars [5, 24]. Despite this, our MR research did not find a causal association between these inflammatory factors—specifically IL‐1β, interleukin 1 receptor‐like 1, MCP1, RANTES/CCL5, TNFα, IL‐8, and IL‐18—and the development of hypertrophic scars. The absence of significant associations in our study does not automatically suggest that these factors are not involved in hypertrophic scar formation. Instead, it may reflect the multifaceted nature of inflammatory processes and the possibility that the genetic variants examined may not encompass the full spectrum of inflammatory pathways involved in the pathogenesis of scars. This discrepancy underscores the need for further research to explore the comprehensive role of inflammation in scar development.

While our study benefits from a robust epidemiological design and thorough sensitivity analyses, it is not without limitations. The reliance on publicly available genome‐wide association study data, predominantly from individuals of European ancestry, may affect the generalizability of our results to other ethnicities. Additionally, the use of a relaxed significance threshold for IV selection, while expanding the candidate pool, could introduce biases. The static nature of genetic information in MR studies might also overlook dynamic changes in protein expression influenced by age, gender, or other physiological factors, which could significantly impact disease risk.

In summary, our MR investigation offers compelling evidence for a causal association between CTACK and the development of hypertrophic scars. This discovery enriches our grasp of the intricate pathophysiological processes that lead to hypertrophic scar formation and could guide the creation of more precise therapeutic interventions. It is imperative that subsequent studies validate these results across diverse populations and delve into the biological underpinnings connecting inflammatory mediators to the pathogenesis of hypertrophic scars.

## Author Contributions

Yanqi Li and Yankun Zhang carried out the studies, participated in collecting data, and drafted the manuscript. Yanqi Li and Wanchao Wang, and Yuge Wang performed the statistical analysis and participated in its design. Yanqi Li and Yuge Wang, along with Hongmei Ai, participated in the acquisition, analysis, or interpretation of data and drafted the manuscript. All authors read and approved the final manuscript.

## Ethics Statement

The authors have nothing to report.

## Consent

The authors have nothing to report.

## Conflicts of Interest

The authors declare no conflicts of interest.

## Supporting information


**Table S1.** Overview of the source of outcome and exposure data.

**Table S2.** SNPs identified in this MR analysis.

Appendix S1.

## Data Availability

All data generated or analyzed during this study are included in this published article.
