# Supplementary material for: Causal Association Between Inflammatory Factors and Hypertrophic Scar: A Two‐Sample Mendelian Randomization Study
Source: J Cosmet Dermatol. 2025 Feb 27;24(3):e70073. doi: 10.1111/jocd.70073 (PMC11866262; doi:10.1111/jocd.70073)
Supplement: Supplementary file 1 — Table S1. Overview of the source of outcome and exposure data. [file JOCD-24-e70073-s001.docx]

**Supplementary table 1 Overview of the source of outcome and exposure data**

| **Trait** | **GWAS ID** | **Samples（case/control）** |
| --- | --- | --- |
| Hypertrophic scar | L12_HYPETROPHICSCAR | 1641/385509 |
| IL-1b | ebi-a-GCST004448 | 3309 |
| interleukin 1 receptor-like 1 | ebi-a-GCST90019444 | 10708 |
| MCP1, Monocyte chemoattractant protein-1 | ebi-a-GCST004438 | 8293 |
| RANTES/CCL5 | ebi-a-GCST004431 | 3421 |
| TNFa, Tumor necrosis factor alpha | ebi-a-GCST004426 | 3454 |
| IL-8 | ebi-a-GCST004445 | 3526 |
| IL-18 | ebi-a-GCST004441 | 3636 |
| CTACK/CCL27 | ebi-a-GCST004420 | 3631 |
